# The Impact of Exercise on C-reactive Protein Levels in Hypertensive Patients: A Systematic Review

**DOI:** 10.7759/cureus.68821

**Published:** 2024-09-06

**Authors:** Tatchaya Kanthajan, Manorama Pandey, Osamah AlQassab, Chithra Sreenivasan, Aneri Parikh, Aida J Francis, Marcellina Nwosu

**Affiliations:** 1 Internal Medicine, California Institute of Behavioral Neurosciences & Psychology, Fairfield, USA; 2 Clinical Research, California Institute of Behavioral Neurosciences & Psychology, Fairfield, USA

**Keywords:** c-reactive protein (crp), cardiovascular risks, exercise, physical fitness, inflammation, hypertension

## Abstract

Hypertension, defined as persistently elevated blood pressure, is a prevalent chronic condition and a significant global health issue, closely linked to cardiovascular complications, with inflammation being one of the underlying mechanisms. In hypertensive patients, C-reactive protein (CRP), an inflammatory marker, is often elevated and associated with increased cardiovascular risk. Alongside pharmacotherapy, exercise is recommended as a non-pharmacological approach to managing hypertension, with evidence suggesting that exercise can also reduce inflammation. This study examines the impact of exercise on CRP levels in hypertensive patients. Fourteen studies focusing on exercise interventions and physical fitness related to CRP in individuals with high blood pressure were identified through an extensive search of PubMed, PubMed Central, ScienceDirect, Cochrane Library, and Google Scholar. The findings indicated that most studies involving aerobic exercise consistently demonstrated reductions in CRP levels among hypertensive patients, with significant effects observed under supervised conditions, and additional benefits seen when combined with dietary control. Resistance training showed mixed results, with significant reductions in CRP observed primarily in longer-term interventions. Combined exercise training, incorporating both aerobic and resistance elements, effectively reduced CRP levels and improved cardiovascular health markers. Physical fitness assessments, such as a bicycle exercise test to exhaustion, revealed a relationship between physical fitness and decreased CRP levels. Therefore, regular, consistent aerobic and combined training, as well as prolonged resistance exercise, significantly reduce CRP levels in hypertensive patients, highlighting exercise's role as a non-pharmacological strategy for managing hypertension through the reduction of inflammation. Further research is essential to validate these findings and investigate the underlying mechanisms and differential effects of various exercise modalities.

## Introduction and background

Hypertension, clinically referred to as elevated blood pressure, is a prevalent chronic medical condition worldwide. The World Health Organization (WHO) identifies it as a leading cause of premature death, estimating that approximately 1.28 billion individuals between the ages of 30 and 79 suffer from hypertension globally. This condition leads to various complications, particularly cardiovascular disease [1]. The widely accepted diagnostic threshold for hypertension is a systolic blood pressure (SBP) of 140 mmHg or higher or a diastolic blood pressure (DBP) of 90 mmHg or higher. However, recent guidelines and studies advocate using an SBP of 130 mmHg and a DBP of 80 mmHg to enable earlier and more effective intervention, helping prevent cardiovascular outcomes and mortality [2].

Inflammation is recognized as one of the underlying mechanisms in the pathogenesis of hypertension. Both innate and adaptive immune cells contribute by producing reactive oxygen species, cytokines, and adhesion molecules. These factors lead to vascular remodeling, endothelial dysfunction, and consequent neurohormonal dysregulation, ultimately resulting in the development of hypertension [3-5]. C-reactive protein (CRP) is commonly used as a marker to evaluate inflammation. It is an acute-phase protein predominantly produced by hepatocytes, with smaller quantities synthesized by vascular smooth muscle cells and macrophages. Additionally, CRP is produced by atherosclerotic lesions [6,7]. Studies have shown that CRP levels are significantly elevated in hypertensive patients compared to those with normal blood pressure, indicating an inflammatory component in hypertension and its relevance to arterial stiffness and end-organ damage. Moreover, CRP can also serve as a predictor for cardiovascular health, and in individuals with normal blood pressure, elevated CRP levels can predict the future development of hypertension [5,8,9]. As a result, CRP testing has become a tool for evaluating the effectiveness of treatments or interventions aimed at reducing cardiovascular outcomes. A more sensitive assay known as high-sensitivity CRP (hs-CRP) can detect CRP levels at lower levels than standard CRP. Elevated CRP or hs-CRP levels can identify patients at higher risk of cardiovascular events and assess the impact of various therapies on inflammation and cardiovascular health [10-13].

Besides anti-inflammatory medications, exercise has been found to provide significant benefits for immune function and reduce inflammation. It can lower CRP levels and improve cardiovascular health through various mechanisms, including enhanced insulin sensitivity, improved blood pressure regulation, enhanced endothelial function, and reduced blood viscosity [14,15]. Furthermore, according to the 2020 International Society of Hypertension Global Hypertension Practice Guidelines, regular exercise is recommended as a nonpharmacological treatment for hypertension. Moderate-intensity aerobic activities, such as swimming or jogging for 30 minutes on 5-7 days a week or high-intensity interval training (HIIT), can effectively manage hypertension. Additionally, incorporating strength training exercises 2 to 3 times a week can further help reduce blood pressure [16].

Therefore, this systematic review aims to examine the effect of exercise on CRP levels, specifically in hypertensive patients, who are more prone to higher inflammation and elevated CRP levels. This review will synthesize current evidence, providing insights into how physical activity can serve as a non-pharmacological approach to managing hypertension and reducing inflammation. This effort will contribute to a broader understanding of exercise as a therapeutic strategy, ultimately improving cardiovascular health outcomes.

## Review

Methods

A PICO (Population, Intervention, Comparison, and Outcome) framework was established to structure the methods section. The population (P) includes hypertensive individuals with SBP above 130 mmHg and DBP above 80 mmHg. The primary intervention (I) under investigation is the impact of exercise on CRP levels in these individuals. This will be compared (C) to the CRP levels of hypertensive patients who do not exercise. The primary outcome (O) is to determine whether exercise affects CRP levels in hypertensive individuals. This study adheres to the 2020 Preferred Reporting Items for Systematic Reviews and Meta-Analyses (PRISMA) guidelines [17]. A computerized search of the literature was conducted between April 2024 and May 2024.

Search Strategy

PubMed, PubMed Central (PMC), ScienceDirect, Cochrane, ClinicalTrials, and Google Scholar were used as research databases and search engines. Medical Subject Headings (MeSH) were created in the PubMed database to conduct an advanced search, as indicated in Table 1. We combined the pertinent concepts with specific keywords using a MeSH strategy and then integrated the MeSH terms and keywords for the three concepts using AND for an advanced research strategy.

**Table 1:** Search strategy using MeSH terms and keywords MeSH: Medical Subject Headings

Concepts	Keywords	Combined MeSH with Keywords using OR
Exercise	Exercise	Exercise OR ( "Exercise/adverse effects"[Majr] OR "Exercise/classification"[Majr] OR "Exercise/history"[Majr] OR "Exercise/immunology"[Majr] OR "Exercise/physiology"[Majr] OR "Exercise/psychology"[Majr] OR "Exercise/standards"[Majr] OR "Exercise/statistics and numerical data"[Majr] OR "Exercise/trends"[Majr] )
C Reactive Protein	C Reactive Protein	C Reactive Protein OR ( "C-Reactive Protein/adverse effects"[Majr] OR "C-Reactive Protein/analysis"[Majr] OR "C-Reactive Protein/chemistry"[Majr] OR "C-Reactive Protein/history"[Majr] OR "C-Reactive Protein/immunology"[Majr] )
Hypertension	Hypertension	Hypertension OR ( "Hypertension/blood"[Majr] OR "Hypertension/classification"[Majr] OR "Hypertension/diagnosis"[Majr] OR "Hypertension/diagnostic imaging"[Majr] OR "Hypertension/diet therapy"[Majr] OR "Hypertension/epidemiology"[Majr] OR "Hypertension/history"[Majr] OR "Hypertension/immunology"[Majr] OR "Hypertension/metabolism"[Majr] OR "Hypertension/physiopathology"[Majr] OR "Hypertension/prevention and control"[Majr] OR "Hypertension/rehabilitation"[Majr] OR "Hypertension/therapy"[Majr] )

For Google Scholar, we used the query ‘allintitle: "Exercise" "Hypertension OR Hypertensive" "CRP OR inflammation OR inflammatory"’. For ClinicalTrials.gov, ScienceDirect, and the Cochrane Library, we used the search terms ‘Exercise AND CRP AND Hypertension’. Filters were applied exclusively to ScienceDirect, where we limited the results to review articles, research articles, English-language publications, open access, and open archive sources.

Selection and Data Collection Process

All relevant articles were initially collected, with duplicates subsequently removed. The remaining articles were then evaluated based on their titles and abstracts, followed by a full-text review to exclude irrelevant studies. The selected articles were then subjected to quality evaluation using appropriate assessment tools.

Inclusion Criteria

This systematic review focuses on individuals of all age groups, including both children and adults, with high blood pressure. Therefore, we included studies reporting data on participants diagnosed with hypertension or high blood pressure, defined as SBP over 130 mmHg and DBP higher than 80 mmHg, and reporting on exercise or physical fitness and CRP levels. Only studies published in full text and the English language were included.

Exclusion Criteria

The exclusion criteria for this systematic review include studies that do not focus on individuals with high blood pressure, report CRP levels, or provide data on exercise or physical fitness. Additionally, studies conducted on animals rather than human subjects, those published in languages other than English, those published before the year 2005, and studies based on grey literature are excluded.

Quality Assessment

**Figure 1:**
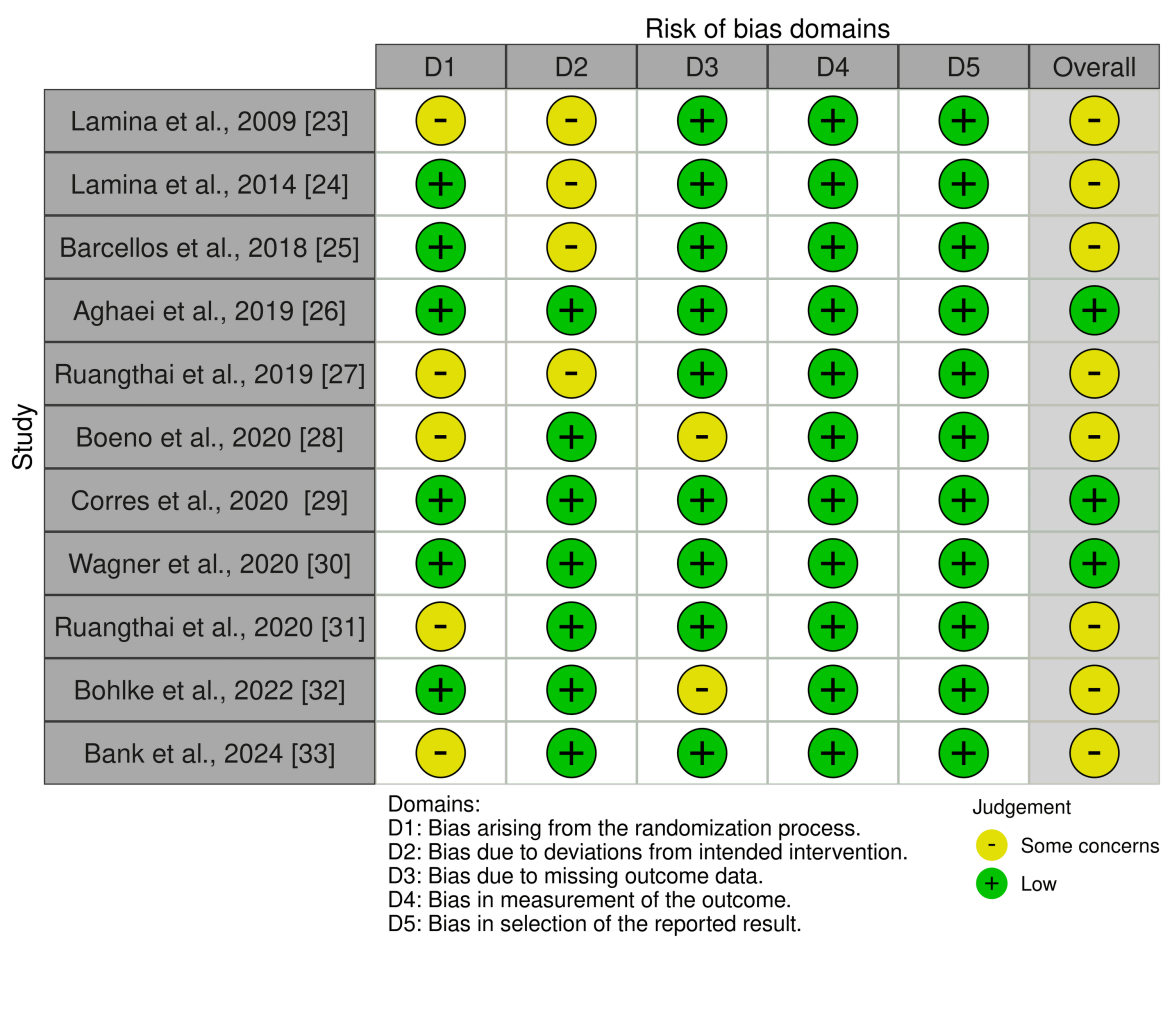
Version 2 of the Cochrane risk-of-bias tool for randomized trials (RoB 2) for quality assessment of included randomized controlled trials

This systematic review incorporated randomized controlled trials (RCTs), cohort studies, and cross-sectional studies. Quality appraisal tools, including Version 2 of the Cochrane Risk-of-Bias Tool for Randomized Trials (RoB 2) for randomized controlled trials and the Newcastle-Ottawa Scale (NOS) for cohort and cross-sectional studies, were used to assess the risk of bias [18,19]. Only articles that satisfied at least 60% of the criteria were selected. The results of the RoB 2 and NOS assessments of the included final studies in this review are shown in Table 2 and Figure 1, respectively.

**Table 2:** The Newcastle-Ottawa Scale (NOS) for quality assessment of included cohort and cross-sectional studies

Study Reference	Study Design	Representativeness of the Sample	Selection of the Non-Exposed Cohort	Ascertainment of Exposure	Demonstration That Outcome of Interest Was Not Present at the Start of Study	Comparability of Cohorts on the Basis of the Design or Analysis	Assessment of Outcome	Was Follow-Up Long Enough for Outcomes to Occur	Adequacy of Follow-Up of Cohorts	Total Score
Lammers et al., 2020 [20]	Prospective cohort	1	1	1	1	1	1	1	1	8/9
Hjelstuen et al., 2006 [21]	Cross-sectional	1	1	0	1	1	1	1	1	6/10
Corres et al., 2018 [22]	Cross-sectional	1	1	0	2	1	1	1	1	7/10

Results

**Figure 2:**
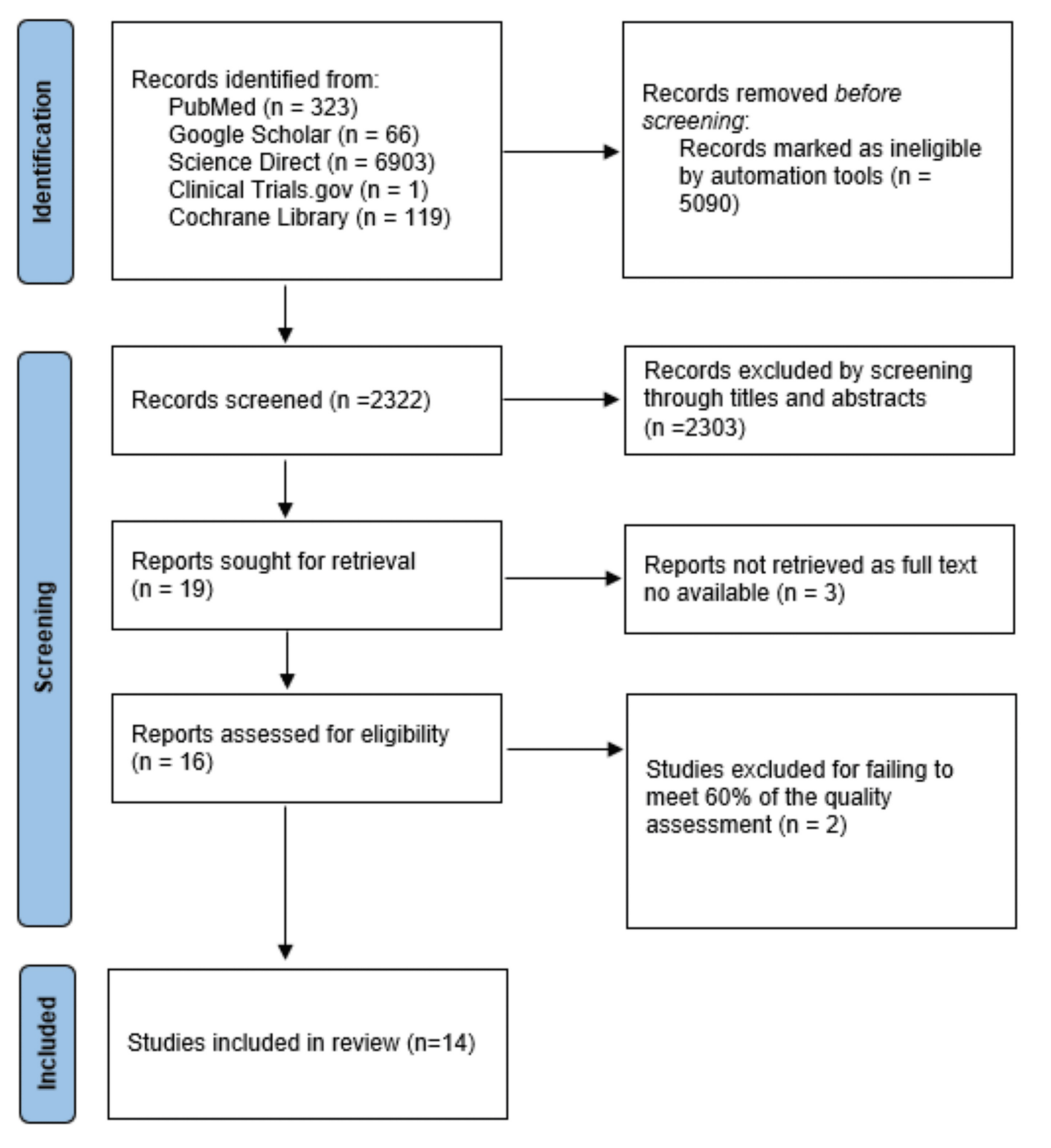
PRISMA flowchart PRISMA: Preferred Reporting Items for Systematic Review and Meta-Analysis

To identify relevant studies, we conducted an electronic search across five databases. Initially, we retrieved 7,412 papers, of which 5,090 were removed using automation tools on ScienceDirect. After applying the inclusion and exclusion criteria and screening by titles and abstracts, we narrowed the selection to 16 full-text articles. Subsequently, quality assessment tools were used to evaluate the risk of bias in these studies. However, two studies did not meet 60% of the quality assessment criteria and were therefore excluded, resulting in 14 articles that met the required standards. Ultimately, we included these 14 papers in the study. The search methodology employed in this review is illustrated in the PRISMA flowchart shown in Figure 2.

After assessing for potential biases, we summarized the data. Table 3 presents the study purpose, number of participants, study type, results, and conclusions for further discussion. Details of the exercise interventions are summarized in Table 4.

**Table 3:** Summary of final articles CRP: C-reactive protein; hs-CRP: high-sensitivity C-reactive protein; ED: erectile dysfunction; P: p-value; RCT: randomized controlled trial; CKD: chronic kidney disease; GFR: glomerular filtration rate; CBT: combined endurance and strength training; RET: resistance exercise training; ET: endurance training; ST: strength training; CRF: cardiorespiratory fitness; LET: land-based exercise training; WET: water-based exercise training; HIIT: high-intensity interval training; IL-1: interleukin 1; IL-6: interleukin 6; HRQoL: health-related quality of life; SupExT: supervised aerobic exercise training; AC: attentional control; MUO: metabolically unhealthy obese

Author and Year of Publication	Exercise/ Intervention	Participants		Duration	Type of Study	Change in CRP levels	Conclusion
		Total Number	Characteristics				
Lammers et al., 2020 [20]	Resistance training program	59 participants (31 in the hypertensive group, 28 in the normotensive group)	Sedentary middle-aged women aged 40-60 years, with and without a diagnosis of hypertension	6 months	Prospective cohort study	CRP significant reduction in the hypertensive group (1.22 ± 0.1 to 0.95 ± 0.1 mg/l).	Resistance training significantly reduced blood pressure, inflammatory markers, and serum adenosine triphosphate levels and improved anti-inflammatory cytokine levels and purinergic enzyme activities in hypertensive women.
Hjelstuen et al. 2006 [21]	Assessment of physical activity through the HYRIM Physical Activity Questionnaire and measurement of physical fitness via a bicycle exercise test to exhaustion	177 participants	Sedentary men aged 40-74 years with drug-treated hypertension	-	Cross-sectional study	hs-CRP demonstrated a significant inverse relationship with physical fitness, independent of major cardiovascular risk factors (P = 0.017), but showed no association with physical activity.	The study suggests that maintaining good fitness status and engaging in low-intensity activities benefit vessel wall inflammation in sedentary men with hypertension.
Corres et al. 2018 [22]	Assessment of cardiorespiratory fitness (CRF)	214 participants (138 men, 76 women)	Overweight/obese adults with primary hypertension, aged 40-74 years, BMI > 25 kg/m²	-	Cross-sectional study	CRP has a negative association with CRF in unadjusted models (P<0.001), but this association is not significant after adjusting for other factors (P=0.090 and 0.084).	The study suggests that lower CRF is related to a worsened biochemical profile in overweight or obese hypertensive patients.
Lamina et al., 2009 [23]	Aerobic exercise: Continuous training program on a bicycle ergometer	43 participants (22 in the continuous training group, 21 in the control group)	Hypertensive men with erectile dysfunction, aged 50-70 years	8 weeks	RCT	CRP significant reduction in exercise group (P < 0.05)	Continuous training significantly reduced blood pressure and CRP levels and improved ED and aerobic capacity in men with ED with hypertension.
Lamina et al., 2014 [24]	Aerobic exercise: Interval training program on a bicycle ergometer	245 participants (140 in the interval training group, 105 in the control group)	Hypertensive men with essential hypertension, aged 45-70 years	8 weeks	RCT	CRP significant reduction in the interval group (P = 0.000)	The interval training program significantly reduced blood pressure, CRP levels, and adiposity indices while improving aerobic capacity in hypertensive black African men.
Barcellos et al., 2018 [25]	Supervised aerobic and resistance training	150 participants (76 in the intervention group, 74 in the control group).	Non-diabetic patients with hypertension and chronic kidney disease, aged 18 years and older, with a mean age of 65 years in both groups	16 weeks	RCT	hs-CRP significant reduction in the exercise group compared to the control group (−6.7 mg/L, 95% CI: −11.7 to −1.8)	Combined aerobic and resistance training significantly reduced hs-CRP and fasting blood glucose levels and improved functional capacity in hypertensive patients with CKD stages 2-4.
Aghaei et al., 2019 [26]	High-intensity interval training (HIIT)	30 participants (10 in the short-duration HIIT group, 10 in the long-duration HIIT group, and 10 in the control group)	Middle-aged hypertensive individuals on valsartan, losartan, or amlodipine	10 weeks	RCT	No significant change	HIIT improves systolic blood pressure and inflammatory markers such as IL-6 and IL-10, but does not significantly affect CRP levels.
Ruangthai et al., 2019 [27]	Combined endurance and strength training (CBT), endurance training (ET), and strength training (ST)	67 participants (16 in the endurance training group, 14 in the strength training group, 17 in the combined endurance and strength training group, and 20 in the control group)	Hypertensive older adults, aged over 60 years	12 weeks	RCT	CBT significantly decreased hs-CRP in supervised training, while ET and ST alone did not show significant changes in CRP.	CBT significantly reduced blood pressure and improved antioxidant capacity, oxidative stress, and inflammation. Participants in the CBT group demonstrated better adherence and attendance, particularly under supervision, resulting in sustained improvements in both SBP and DBP.
Boeno et al., 2020 [28]	Supervised aerobic training on a treadmill and supervised dynamic resistance training	42 participants (15 in the aerobic training group, 15 in the resistance training group, 12 in the control group)	Middle-aged hypertensive individuals on at least one antihypertensive medication, aged 30-59 years	12 weeks	RCT	Aerobic exercise significantly decreased CRP (−1.0 ± 1.7 mg/dL, P<0.05) and other inflammation markers.	Both aerobic and resistance training effectively lower blood pressure and improve endothelial function. Aerobic exercise also significantly reduces inflammation.
Corres et al., 2020 [29]	Supervised aerobic exercise training (SupExT) combined with a hypocaloric diet	177 participants (SupExT Group: 134, attentional control (AC) Group: 43)	Physically inactive, overweight/obese individuals with primary hypertension	16-week intervention with a 6-month follow-up	RCT	Significant reduction in CRP levels post-intervention in SupExT group (Δ = −1.5 mg/L) and AC group (Δ = −1.0 mg/L). Slight increase in CRP levels during follow-up but maintained reduction in SupExT group (Δ = −1.2 mg/L)	The intervention significantly improved the cardiometabolic profile of MUO individuals, but improvements were not fully maintained after 6 months of unsupervised follow-up, highlighting the need for ongoing supervised programs.
Wagner et al., 2020 [30]	Aerobic cardio training combined with caloric reduction	68 participants (18 in the cardio training and caloric reduction group, 28 in the cardio training Alone group, 22 in the control group)	Sedentary prehypertensive and mildly hypertensive individuals (mean age ± SEM: 45 ± 1 years; mean BP: 141/84 ± 1/1 mmHg)	12 weeks	RCT	Significant decrease in CRP levels with caloric reduction (p = 0.018) Decrease in CRP levels in cardio training alone (not statistically significant) No significant change in CRP levels in control group	Combined intervention resulted in significant reductions in CRP levels, indicating the potential benefits of combined exercise and dietary interventions for reducing inflammation in hypertensive individuals. Aerobic cardio training alone resulted in a decrease in CRP levels, but the change was not statistically significant. No significant change in CRP levels was observed in the control group.
Ruangthai et al., 2020 [31]	Combined aerobic, resistance, and stretching exercise training: Land-Based Exercise Training (LET): 12 weeks supervised, then 12 weeks self-supervised; Water-Based Exercise Training (WET): 12 weeks supervised, then 12 weeks self-supervised	53 participants (17 in the land-based exercise group, 16 in the water-based exercise group, 20 in the control group)	Hypertensive older adults, aged over 60 years	24 weeks	RCT	After supervised training, both LET and WET significantly decreased hs-CRP. In self-supervised exercise, only WET decreased hs-CRP.	Both LET and WET significantly improved blood pressure, antioxidant capacity, oxidative stress, inflammatory markers, physical capability, and well-being in elderly individuals with hypertension.
Bohlke et al., 2022 [32]	16-week aerobic and resistance training (follow-up from Barcellos et al., 2018 [25])	150 participants (76 in the intervention group, 74 in the control group)	Hypertensive patients with CKD stages 2-4, aged 65 years on average)	16 weeks of supervised training, followed by 3 years post-intervention	RCT with long-term follow-up	No significant long-term effects on hs-CRP levels	The exercise intervention had no significant long-term effects on survival, GFR, or HRQoL, indicating the need for continuous exercise interventions for lasting health benefits.
Banks et al., 2024 [33]	Resistance exercise training (RET) intervention	42 participants (15 in the aerobic training group, 15 in the resistance training group, and 12 in the control group)	Middle-aged hypertensive individuals	9 weeks	RCT	RET did not result in significant changes in CRP levels.	RET significantly decreased blood pressure and improved vascular endothelial function without affecting CRP levels.

**Table 4:** Exercise intervention details from included research studies CBT: combined endurance and strength training; LET: land-based exercise training; WET: water-based exercise training; HIIT: high-intensity interval training

	Exercise Intervention	Duration	Frequency	Session Duration	Type of Exercise
Lammers et al., 2020 [20]	Supervised resistance training	6 months	2 times per week	45-60 minutes	Resistance training
Hjelstuen et al., 2006 [21]	Bicycle exercise test to exhaustion	Test duration unknown	Single test	Until exhaustion	Cardiorespiratory fitness assessment
Lamina et al., 2009 [23]	Continuous training program on a bicycle	8 weeks	3 times per week	45-60 minutes	Aerobic exercise
Lamina et al., 2014 [24]	Interval training program on a bicycle ergometer	12 weeks	3 times per week	45-60 minutes	Interval training on a bicycle ergometer
Barcellos et al., 2018 [25]	Combined aerobic and resistance training	16 weeks	3 times per week	60 minutes	Aerobic + resistance training
Aghaei et al., 2019 [26]	High-intensity interval training (HIIT)	12 weeks	Varied intervals	30-40 minutes	High-intensity interval training (HIIT)
Ruangthai et al., 2019 [27]	Combined Endurance and Strength Training (CBT)	12 weeks supervised + 12 weeks self-supervised	3 times per week	Unspecified	Endurance and Strength Training
Boeno et al., 2020 [28]	Dynamic resistance training	12 weeks	3 times per week	Unspecified	Resistance training
Corres et al., 2020 [29]	Supervised aerobic exercise combined with hypocaloric diet	16 weeks + 6 months follow-up	3 times per week	60 minutes	Aerobic exercise + hypocaloric diet
Wagner et al., 2020 [30]	Aerobic cardio training combined with caloric reduction	12 weeks	3 times per week	45-60 minutes	Aerobic cardio training
Ruangthai et al., 2020 [31]	Combined Land-Based and Water-Based Exercise Training (LET and WET)	12 weeks supervised + 12 weeks self-supervised	3 times per week	Unspecified	Land-Based and Water-Based Exercise Training
Bohlke et al., 2022 [32]	Combined aerobic and resistance training	16 weeks + 3 years follow-up	3 times per week	60 minutes	Aerobic + resistance training
Banks et al., 2024 [33]	Resistance exercise training	9 weeks	3 times per week	45-60 minutes	Resistance training

Discussion

Aerobic Exercise

Aerobic exercise refers to sustained physical activity where the blood continuously delivers oxygen to the working muscles. This type of metabolism occurs during low-intensity, long-duration exercise through rhythmic, continuous activities involving large muscle groups. These exercises use aerobic metabolism to generate energy or adenosine triphosphate (ATP) from amino acids, carbohydrates, and fats and are well known to help with cardiovascular disease [34]. Our review has demonstrated that aerobic exercise is effective in reducing CRP levels. For example, a study by Lamina et al. in 2009 observed a significant decrease in CRP levels in hypertensive men with erectile dysfunction who participated in a continuous cycling training program, compared to those in a control group [23]. Similarly, in 2014, Lamina et al. found that an interval training program on a bicycle ergometer significantly reduced CRP levels in the interval group compared to the control group. This study also highlighted the efficacy of interval training in lowering blood pressure and inflammation while improving fitness and reducing adiposity [24]. Supporting these findings, Boeno et al. demonstrated that while both aerobic and resistance training significantly reduced blood pressure and improved endothelial function, only aerobic training led to a significant decrease in markers of inflammation. These findings underscore the additional benefits of aerobic exercise in reducing systemic inflammation, although resistance training also offers essential health benefits [28]. HIIT, a type of aerobic exercise characterized by alternating periods of intense activity with intervals of passive or active recovery, has also been investigated. Aghaei et al. found that HIIT did not significantly affect CRP levels, despite its positive effects on blood pressure and inflammatory markers such as IL-6 and IL-10. The authors hypothesized that the lack of impact on CRP may be due to insufficient weight loss in the study participants, as CRP is also produced by macrophages in adipose tissue [26]. This corresponds with findings from Corres et al. and Wagner et al., which highlight the importance of combining aerobic exercise with dietary modifications, particularly caloric reduction. These studies demonstrated significant reductions in CRP levels when aerobic exercise was paired with dietary interventions in hypertensive and at-risk populations. This combination not only lowered CRP levels but also promoted weight loss, further contributing to improved cardiometabolic health [29,30]. However, these benefits require consistency and ongoing efforts, as evidenced by Corres et al., who found that supervised aerobic exercise and diet interventions significantly reduced CRP levels and improved the cardiometabolic profile. Although CRP levels after six months of unsupervised follow-up in the supervised intervention group remained lower than pre-intervention levels, they were still higher than during the intervention period. The improvement in CRP was not fully maintained after six months of unsupervised follow-up, indicating the need for ongoing, supervised exercise and diet programs to sustain health benefits [29]. In summary, most of the included RCTs investigating aerobic exercise interventions found that aerobic exercise significantly reduces CRP levels in hypertensive patients, but consistency and regularity are essential for maintaining this anti-inflammatory effect.

Resistance Exercise

Resistance exercise is a type of physical activity that enhances muscular strength and endurance by challenging muscles to work against a weight or external force. It causes microscopic damage to muscle fibers, triggering repair processes that involve satellite cell activation and muscle protein synthesis. This leads to muscle hypertrophy and increased strength, contributing to overall physical fitness and health [35]. Studies included in this review indicate that resistance exercise may decrease CRP levels, particularly with longer intervention periods. As evidenced by Banks et al., no significant changes in CRP levels were observed in the resistance exercise training (RET) group compared to the control group after a nine-week RET intervention [33]. Similarly, Boeno et al. reported no significant differences in CRP levels after 12 weeks of supervised dynamic resistance training conducted three days per week [26]. Conversely, Lammers et al. found a significant reduction in CRP levels in the hypertensive group after six months of supervised resistance training [20]. The lack of significant change in CRP levels in the studies with nine-week and 12-week interventions, compared to the significant decrease observed in the six-month study, suggests that the anti-inflammatory effects of RET may not be evident in the short term or may require different markers of inflammation for assessment.

Combined Exercise Training

The findings from the included studies on combined exercise interventions provide compelling evidence for the efficacy of mixed aerobic and resistance training in reducing CRP levels in hypertensive patients, highlighting the potential of these interventions in managing inflammation. Moreover, consistency and continuity are crucial for maintaining the long-term benefits of exercise. As demonstrated by Barcellos et al., a 16-week supervised aerobic and resistance training program led to a significant decrease in hs-CRP levels in the exercise group compared to the control group, illustrating the benefits of combined exercise training in reducing inflammation and improving physical fitness in hypertensive patients with chronic kidney disease (CKD) [25]. However, maintaining long-term benefits requires ongoing commitment, as shown by Bohlke et al. who explored the long-term effects of the study by Barcellos et al. and found that while initial benefits were observed, there were no significant long-term effects on CRP levels after three years [25,32]. This is consistent with findings from Ruangthai et al. in 2019, who conducted an RCT evaluating the effects of combined endurance and strength training (CBT). The study included 12 weeks of supervised training followed by 12 weeks of self-supervised training. They observed a significant decrease in hs-CRP levels in the CBT group after the supervised training period, but no significant changes during the self-supervised period. The lower adherence and questionable intensity of exercise during the self-supervised period likely contributed to this outcome [27]. This study highlights the benefits of combined endurance and strength training for improving cardiovascular health and reducing oxidative stress and inflammation in elderly individuals with hypertension while also highlighting that inconsistency in intensity and adherence may lead to a lack of positive effects that would otherwise be expected. Similarly, in another RCT by Ruangthai et al. in 2020, the effects of combined land-based exercise training (LET) and water-based exercise training (WET) were investigated, incorporating aerobic, resistance, and stretching exercises. The study included 12 weeks of supervised training followed by 12 weeks of self-supervised training. Results showed a significant decrease in hs-CRP concentration in both the LET and WET groups after the supervised training period, with a continued significant decrease in the WET group after the self-supervised period [31]. This study demonstrates the benefits of both water- and land-based combined exercise training for improving cardiovascular health, reducing oxidative stress and inflammation, enhancing functional fitness, and improving quality of life in hypertensive older adults. Water-based exercise may offer additional advantages by motivating older adults to adhere to and maintain a regular exercise routine due to its lower impact and reduced fear of falling. In summary, the consistent findings across these studies underscore the importance of a multifaceted exercise approach in managing hypertension and its associated inflammatory markers. Combined exercise training, incorporating both aerobic and resistance elements, effectively reduces inflammation as measured by CRP levels in hypertensive patients. The sustained benefits observed in supervised exercise and water-based exercise programs highlight the importance of regular exercise with consistent intensity and suggest that incorporating such modalities may be particularly beneficial for individuals who have limitations with land-based exercises.

Physical Fitness

In addition to studies directly examining exercise interventions, we included a study that assesses cardiorespiratory fitness (CRF) due to its close relationship with physical activity and exercise. Evidence shows that exercise and physical activity positively affect CRF, with increased exercise intensity and higher levels of physical activity leading to improvements in CRF [36-38]. We also included studies that measure physical fitness and its relation to CRP levels. Consequently, two cross-sectional studies were included, which found that physical fitness is associated with lower CRP levels [21,22]. Hjelstuen et al. assessed physical activity using a questionnaire and measured physical fitness via a bicycle exercise test to exhaustion. Their results showed a significant inverse relationship between hs-CRP levels and physical fitness, independent of major cardiovascular risk factors, though no relationship was found with physical activity [21]. This underscores the benefits of physical fitness on CRP levels in sedentary men with hypertension. Conversely, Corres et al. found that while CRF was significantly associated with lower CRP levels in unadjusted models, this association was not significant in adjusted models, suggesting that other factors may influence the relationship [22]. These findings suggest that a more objective assessment of physical fitness, such as a bicycle exercise test to exhaustion, may provide more robust evidence of the relationship between physical fitness and decreased CRP levels compared to measures based on questionnaires or CRF, which other variables may influence. This highlights the importance of using precise and reliable methods to evaluate physical fitness when examining its impact on exercise or physical fitness and CRP levels.

Mechanism of Exercise in Reducing Inflammation and CRP and Clinical Implications

Exercise mitigates inflammation through several complex mechanisms, including the modulation of cytokine profiles, reduction of adipose tissue inflammation, and enhancement of metabolic health. During acute exercise, interleukin-6 (IL-6) is produced without inducing an inflammatory response and facilitates the increase of anti-inflammatory mediators such as soluble tumor necrosis factor (TNF) receptors and interleukin-1 (IL-1) receptor antagonists. Over time, regular aerobic exercise decreases the size of adipose tissue, which in turn lowers the expression of pro-inflammatory cytokines. Furthermore, the transcriptional co-activator peroxisome proliferator-activated receptor gamma co-activator 1-alpha (PGC-1α) plays a role in maintaining an anti-inflammatory environment [39]. The precise mechanism by which exercise affects CRP levels is not yet fully understood. However, it is hypothesized that similar to its effect on inflammation, exercise reduces CRP levels by decreasing cytokine production, particularly IL-6, which primarily stimulates CRP, along with contributions from IL-1 and TNF-alpha. Exercise reduces these cytokines during physical activity while also enhancing insulin sensitivity, improving endothelial function, and potentially exerting antioxidant effects, eventually leading to a reduction in CRP levels [40-42]. In resistance exercise training, which causes microtrauma to muscles, pro-inflammatory cytokines such as IL-6, TNF-α, and IL-1β are initially released to manage tissue repair. However, with repeated regular resistance training, markers of inflammation and muscle damage are reduced upon repeated exposure to the same exercise stimulus, leading to adaptations [43]. This is supported by the results of three RCTs included in our study, which showed no CRP change in nine-week and 12-week resistance training but reduced CRP levels with six months of training [20,28,33]. This suggests that resistance exercise has a more significant effect in the long term.

Understanding the mechanisms through which exercise reduces inflammation provides insights into how it helps manage hypertension, given that inflammation is a critical factor in its pathogenesis [3]. Beyond the benefit of reducing inflammation, regular exercise helps in other aspects that lead to controlled hypertension. It improves endothelial function, leading to better vasodilation and blood flow, and increases cardiorespiratory fitness, reducing the workload on the heart and lungs, thereby decreasing blood pressure and increasing insulin sensitivity. Additionally, it helps with weight loss [44]. Exercise also aids in mental health by reducing stress and anxiety through various mechanisms, such as the release of endorphins [45]. Overall, these advantages together lead to reducing cardiovascular risk. Thus, regular exercise is a valuable way to manage hypertension with numerous benefits.

The results of our studies can be applied in clinical practice for health promotion and the treatment of hypertension, particularly in reducing inflammation. The studies that implemented aerobic exercise interventions found that reductions in CRP levels were generally achieved with sessions lasting approximately 45-60 minutes, conducted three times per week [23-32], as shown in Table 4. Based on these findings, we suggest that hypertensive patients should be recommended to engage in aerobic exercise for 45-60 minutes at least three times a week to reduce inflammation.

For resistance training, one study with a six-month intervention involving exercise sessions twice a week for 45-60 minutes showed significant reductions in CRP levels [20]. Therefore, similar to aerobic and combined exercise, regular long-term resistance training is likely to be beneficial in reducing inflammation in hypertensive patients.

In combined exercise training, incorporating both aerobic and resistance exercises, all studies included in our review demonstrated a decrease in CRP levels after the combined exercise intervention. In clinical practice, recommending combined exercise regimens may be optimal, as both types of exercise offer distinct benefits beyond inflammation reduction. Even though our review found a significant decrease in CRP levels in only one study with long-term resistance exercise intervention [20], resistance training should still be recommended, as it offers unique benefits such as increased muscle mass, improved bone density, and enhanced muscle strength, which are not as effectively achieved through aerobic exercise alone [46,47].

In addition to maintaining the proper duration and frequency of all types of exercise, consistency should be emphasized, and efforts should be made to identify the most suitable exercise modalities to help patients adhere to their routines and maximize benefits. For example, water-based exercise may be particularly beneficial for elderly patients or those with limitations to land-based exercise [31]. Furthermore, diet control should be recommended in combination with exercise, as it aids in weight loss and ultimately helps reduce inflammation [29,30].

The results from our review of various types of exercise reaffirm the positive effects of exercise in decreasing CRP levels in hypertensive patients by reducing inflammation. This highlights the importance of incorporating exercise into management plans for hypertensive patients. Exercise is an effective non-pharmacological strategy to manage hypertension and improve overall cardiovascular health.

Future research should focus on long-term study designs and investigate the differences in types of exercise training with varying intensities on CRP and other inflammatory markers to gain a deeper understanding of these findings. Additionally, studying larger and more diverse populations will provide greater validation, ensuring the results are more generalizable. This will help identify optimal exercise regimens for managing hypertension by reducing inflammation and, consequently, cardiovascular risk.

Limitations

This systematic review has several limitations. The studies are not homogenous in their measurement of CRP, with some investigating hs-CRP. The included studies exhibit significant heterogeneity in terms of exercise interventions, participant characteristics, and outcome measurements, complicating direct comparisons and synthesis of results. Additionally, some studies have small sample sizes, limiting the generalizability of the findings. Another concern is the risk of bias, primarily related to the blinding of participants and incomplete outcome data, which could potentially overestimate the benefits of exercise. Furthermore, the findings might not specifically apply to specific groups with CRP elevation secondary to other conditions, as we included studies where participants had high blood pressure and focused on the impact of exercise on CRP levels, regardless of their other underlying conditions. Moreover, most studies have short follow-up periods, restricting the ability to assess the prolonged effects of exercise on CRP levels and cardiovascular health. Additionally, our review did not include any systematic reviews or meta-analyses, meaning the highest levels of evidence were not incorporated despite searching across five databases. These limitations should be considered when assessing the findings of this systematic review.

## Conclusions

This systematic review found that various forms of exercise, including aerobic, resistance, and combined exercise, can significantly reduce CRP levels in hypertensive patients. Notably, significant reductions in CRP were observed primarily with longer resistance training, while aerobic and combined exercises also demonstrated significant reductions, particularly during supervised intervention periods. These findings underscore the importance of adherence and consistency in achieving and maintaining these anti-inflammatory effects. Additionally, the inverse relationship between physical fitness and CRP levels further highlights the value of exercise. Therefore, exercise is a valuable non-pharmacological intervention for managing hypertension, as it effectively reduces inflammation and improves cardiovascular health. Encouraging hypertensive patients to engage in regular physical activity is essential for managing their condition. It is also crucial to tailor the exercise regimen, including frequency, duration, and modality, to the individual needs of each patient. Furthermore, dietary control should be recommended to maximize the anti-inflammatory benefits of exercise.
